# Effect of 1.8-mm steep-axis clear corneal incision on the posterior corneal astigmatism in candidates for toric IOL implantation

**DOI:** 10.1186/s12886-020-01456-3

**Published:** 2020-05-06

**Authors:** Xi Li, Xiang Chen, Suhong He, Wen Xu

**Affiliations:** 1 Eye Center, the Second Affiliated Hospital of Zhejiang University, College of Medicine, Hangzhou, 310000 Zhejiang China; 2 Department of Ophthalmology, Shanxi Provincial Cancer Hospital, Affiliated Cancer Hospital of Shanxi Medical University, Taiyuan, China; 3 Department of Ophthalmology, Suichang Hospital of Traditional Chinese Medicine, Suichang, China

**Keywords:** Posterior corneal astigmatism, Cataract surgery, Steep-axis incision

## Abstract

**Background:**

In the present study, we aimed to analyze the effects of cataract surgery using a 1.8-mm steep-axis clear corneal incision (CCI) on the posterior corneal surfaces based on the keratometry from the rotating Scheimpflug imaging device (Pentacam HR) in candidates for toric intraocular lens (IOL) implantation.

**Methods:**

Preoperative and at least 1-month postoperative data measured by Pentacam HR were collected in patients for toric IOL implantation. Surgically induced astigmatism on the posterior cornea (P-SIA) was calculated based on the preoperative and postoperative keratometric data, and the related factors of P-SIA were analyzed.

**Results:**

A total of 60 eyes from 56 patients were enrolled. The preoperative anterior, posterior and total corneal astigmatism was 1.58 ± 0.61 D,0.28 ± 0.22 D and 1.70 ± 0.52 D respectively. The postoperative anterior, posterior and total corneal astigmatism was 1.26 ± 0.68 D, 0.41 ± 0.26 D and 1.30 ± 0.51 D respectively. The astigmatism was significantly decreased on anterior surface (*P*<0.001, paired t-test) and increased on posterior surface (*P*<0.001, paired t-test). The mean of P-SIA calculated by Holladay–Cravy–Koch method was 0.34 ± 0.20 D, with 0.5 D or greater accounting for 26.7%. A statistically significant correlation was observed between the P-SIA and preoperative anterior corneal astigmatism (*r* = 0.29, *P* = 0.024), as well as preoperative posterior corneal astigmatism (*r* = 0.27, *P* = 0.038). Multivariate regression analysis showed the preoperative anterior and posterior corneal astigmatism had a significant effect on P-SIA (F = 7.344, *P* = 0.001).

**Conclusions:**

In candidates for toric IOL implantation with a 1.8-mm steep-axis CCI, the incision caused a significant reduction of the anterior corneal astigmatism but an increase of the posterior corneal astigmatism. P-SIA could not be ignored, and it played a significant role in SIA, especially in cases with higher preoperative anterior or posterior corneal astigmatism.

## Background

The corneal astigmatism greater than 1.0 D, 1.5 D and 2.0 D approximately accounts for 30, 22 and 8% of cataract patients before surgery, respectively [1–3]. Corneal astigmatism may lead to a series of visual discomfort, which will decrease patients’ satisfaction after cataract surgery. No adding on the preexisting astigmatism thereby correcting corneal astigmatism is an important principle in cataract surgery. Nowadays, corneal relaxing incision (CRI), limbal relaxing incision (LRI) and toric intraocular lens (IOL) are used to correct corneal astigmatism, and remarkable results have been achieved [4–7].

The accurate measurements of corneal astigmatism are overwhelmingly important for the correction of corneal astigmatism. In recent years, with the development of the Scheimpflug anterior segment analysis system, the measurement of astigmatism on the posterior cornea has been achieved. Some characteristics about posterior cornea have been revealed [8–11], and it has been widely accepted that the posterior corneal astigmatism is non-ignorable, especially in patients for toric IOL implantation [11–13].

Although the importance of posterior corneal astigmatism has drawn great attention, surgically induced astigmatism (SIA) is calculated based on the keratometric data without taking the real effect of the incision on the posterior corneal surface in previous studies, which may lead to mis-estimation of SIA. For toric IOL implantation especially, accurate SIA estimation is crucial for better postoperative outcomes. However, it still remains controversial whether the cataract incision has a significant influence on the posterior corneal astigmatism [14–16].

In the present study, we aimed to analyze the surgically induced astigmatism on the posterior cornea (P-SIA) in the cataract surgery using a 1.8-mm steep-axis clear corneal incision (CCI) based on the Pentacam HR (Oculus Optikgerate GmbH, Wetzlar, Germany) in candidates for toric IOL implantation and explore the correlation between P-SIA and preoperative parameters (such as preoperative keratometry and astigmatism).

## Methods

This retrospective study adhered to the tenets of the Declaration of Helsinki and had obtained human research ethics approval from the ethics committee of the Second Affiliated Hospital of Zhejiang University. All people voluntarily joined this study with informed consents. Patients, who underwent cataract surgery with a 1.8-mm steep-axis CCI at the Eye Center, the Second Affiliated Hospital of Zhejiang University during March 2017 to December 2018, were enrolled in this study. Patients were included if they met the criteria as follows: (1) patients aged at least 45 years old who were scheduled for cataract surgery; (2) regular corneal astigmatism ≥1.0 D; (3) the imaging quality parameter (QS) of Pentacam HR was “OK”; and (4) follow-up time ≥ 1.0 month. The exclusion criteria were set as follows: (1) irregular corneal astigmatism; (2) corneal scar, corneal degeneration, pterygium invading the optic zone; (3) corneal endothelium count< 1000/mm^2^, corneal endothelial dystrophy, iritis, glaucoma and retinal disease; (4) history of eye surgery, such as corneal refractive surgery, corneal transplantation and retinal surgery; (5) the main incision was expanded or sutures were used during surgery; and (6) some postoperative complications, such as persistent corneal edema, Descemet’s membrane detachment and poor wound healing, and any others that might affect the measurement results of the Pentacam HR during the follow-up period.

Pentacam HR was set to auto mode, scanning was completed within 2 s, and 50 images were taken. The examination was completed in the dark room. The patients were asked to keep their eyeballs still, and the images were automatically captured by the device. Only the measurement with an “OK” reading displayed in the QS window was enrolled; otherwise, this measurement was retried. Eyes that still failed to reach the standard after repetition were excluded.

All surgeries were performed by the same skillful and experienced surgeon Dr. Xu. Standard toric IOL implantation process was performed. Firstly, toirc IOL alignment axis was marked by using a Mendez and a sterile needle. A one-step a 1.8-mm corneal steep-axis CCI was made based on axis of the total corneal astigmatism, followed by the continuous curvilinear capsulorhexis (CCC), phacoemulsification, irrigation/aspiration of the cortex. A TECNIS toric IOLs (AMO, Abbott Park, Illinois, USA) was implanted in the capsular bag without previous enlargement of the main incision and rotated to the target position.

The following data were collected: the patient’s identification number, age, gender, OD/OS, date of surgery, date of follow-up; and the following parameters of the corneal central 3 mm measured by Pentacam HR, both preoperative and postoperative: anterior corneal K_1_ and K_2_, astigmatism and its axis, posterior corneal K_1_ and K_2_, astigmatism and its axis. The corneal astigmatism, both anterior and posterior, was classified as with-the-rule astigmatism (WTR, 60° ~ 120°), against-the-rule astigmatism (ATR, 0 ~ 30°or 150 ~ 180°) and oblique astigmatism (30–60° or 120–150°) according to the astigmatism axis.

P-SIA was calculated according to the preoperative and postoperative corneal curvature parameters (K_1_, K_2_ and astigmatism axis) using the Holladay-Cravy-Koch method [17, 18].

Double-angle plots [19] were performed to show the distribution of total corneal astigmatism and astigmatism in the anterior, posterior corneal surfaces, as well as the P-SIA. The distance between each spot to the original point represented the size of astigmatism, and the angle of each spot was the doubled astigmatism axis.

### Statistical analysis

All statistical analyses were performed using SPSS software version 20.0 (SPSS, Inc., Chicago, IL). Paired t-tests were used to compare the differences between preoperative and postoperative mean astigmatisms. Independent t-test and One-way analysis of variance (ANOVA) were performed for comparisons of different groups. The correlation between P-SIA and parameters was assessed by using Pearson’s correlation analysis. Multivariate regression analysis was performed to assess the association of P-SIA with independent variables, including age, sex, and preoperative keratometric data. Variables were kept in the model if they were associated with a *P* value <0.05 and the overall fit of the model improved as indicated by the change of R^2^, which was used to find the best fitting multivariable model. A *P* value less than 0.05 was considered as statistically significant.

## Results

### Demographics

In the present study, 60 eyes of 56 patients with 1.8-mm steep-axis CCI, including 24 males and 32 females, were enrolled. The age of patients ranged from 43 to 82 years with an average of 58.8 ± 10.7 years. The follow-up period ranged from 4.0 to 13.6 weeks with an average of 8.7 ± 3.4 weeks.

### Anterior corneal astigmatism

The numbers of eyes with WTR astigmatism, ATR astigmatism and oblique astigmatism of the preoperative anterior corneal were 34, 24 and 2, respectively. The number of eyes with the preoperative anterior corneal astigmatism less than 1.5 D accounted for 48.3%, that of ≥1.5 D and < 2.5 D accounted for 43.3%, and that of more than 2.5 D accounted for 8.3% (Table 1).

**Table 1:** Distribution and types of preoperative astigmatism (N, %)

	Anterior corneal (***n*** = 60)		Posterior corneal (***n*** = 60)		Total corneal (***n*** = 60)	
**Distribution**	**< 1.5 D**	29 (48.3%)	**< 0.5 D**	49 (81.7%)	**< 1.5 D**	21 (35.0%)
	**1.5 ~ 2.5 D**	26 (43.3%)	**0.5 D ~ 1.0 D**	11 (18.3%)	**1.5 ~ 2.5 D**	34 (56.7%)
	**≥2.5 D**	5 (8.3%)	**≥1.0 D**	0 (0.0%)	**≥2.5 D**	5 (8.3%)
**Type**	**WTR**	34 (56.7%)	**WTR**	34 (56.7%)	**WTR**	34 (56.7%)
	**ATR**	24 (40.0%)	**ATR**	10 (16.7%)	**ATR**	23 (38.3)
	**oblique**	2 (3.3%)	**oblique**	9 (15.0%)	**oblique**	3 (5.0%)
			**No astigmatism**	7 (11.7%)		

*WTR* with-the-rule astigmatism, *ATR* against-the-rule astigmatism, *D* diopters

**Fig. 1:**
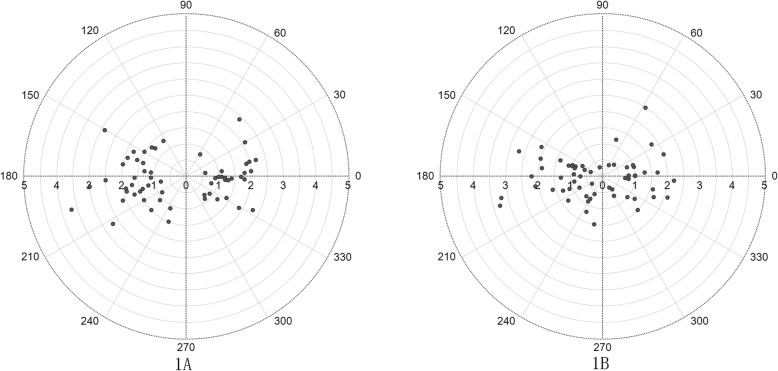
Double-angle plot of the distribution of astigmatism on the anterior corneal surface. **a** is the preoperative anterior corneal astigmatism. **b** is the postoperative anterior corneal astigmatism

The preoperative anterior corneal astigmatism ranged from 0.70 D to 3.70 D with an average of 1.58 ± 0.61 D. The mean preoperative anterior corneal K_1_ was 43.36 ± 1.67 D, and K_2_ was 44.96 ± 1.64 D. The postoperative anterior corneal astigmatism ranged from 0.30 D to 3.30 D, with an average of 1.26 ± 0.68 D. The postoperative K_1_ was 43.32 ± 1.74 D, and K_2_ was 44.61 ± 1.68 D. Paired t-test showed a significant reduction in postoperative anterior corneal K_2_ compared with the preoperative one (*P* < 0.001), and no significant difference was found in postoperative anterior corneal K_1_ and the preoperative one (*P* = 0.395). Paired t-test showed a significant reduction in postoperative anterior corneal astigmatism compared with the preoperative one (*P* < 0.001) (Table 2). Figure 1 shows the preoperative and postoperative astigmatism distribution of anterior cornea. A concentration trend could be found between Fig. 1a and b.

**Table 2:** Preoperative and postoperative corneal astigmatism

variance		Mean ± SD	95%CI	minimum	maximum
**Anterior corneal**	Astigmatism (D)	1.58 ± 0.61	1.42,1.73	0.70	3.70
	K_1_ (D)	43.36 ± 1.67	42.92,43.79	39.20	46.60
	K_2_, (D)	44.96 ± 1.64	44.54,45.38	41.40	48.60
	Astigmatism (D)	1.26 ± 0.68*	1.08,1.44	0.30	3.30
	K_1_, (D)	43.32 ± 1.74	42.87,43.77	39.20	46.90
	K_2_, (D)	44.61 ± 1.68	44.18,45.05	40.50	48.00
**Posterior corneal**	Astigmatism (D)	0.28 ± 0.22	0.22,0.33	0.00	0.80
	K_1_ (D)	−6.28 ± 0.25	−6.35, −6.22	−6.80	−5.80
	K_2_, (D)	−6.55 ± 0.27	−6.62, − 6.48	−7.20	−6.00
	Astigmatism (D)	0.41 ± 0.26*	0.35,0.48	0.00	1.10
	K_1_, (D)	−6.33 ± 0.28*	−6.41, − 6.26	−5.80	−6.90
	K_2_, (D)	−6.75 ± 0.37*	−6.85, − 6.66	−6.10	−7.70

The shaded part represents preoperative corneal astigmatism; and the non-shaded parts represent postoperative corneal astigmatism. Asterisk* represents a significant difference (*P* < 0 .05) compared with the preoperative astigmatism. *SD* standard deviation, *CI* confidence interval, *D* diopters, *K*_*1*_ keratometric value at the flattest corneal meridian, *K*_*2*_ keratometric value at the steepest corneal meridian

### Posterior corneal astigmatism

The numbers of eyes with WTR astigmatism, ATR astigmatism and oblique astigmatism of the preoperative posterior corneal were 34, 10 and 9, respectively, of which WTR astigmatism accounted for 56.7%. There were 49 eyes with a preoperative posterior corneal astigmatism less than 0.5 D, accounting for 81.7% (including seven eyes with an astigmatism of 0 D), and there were 11 eyes with an astigmatism of more than 0.5 D and less than 1.0 D, accounting for 18.3% (Table 1).

**Fig. 2:**
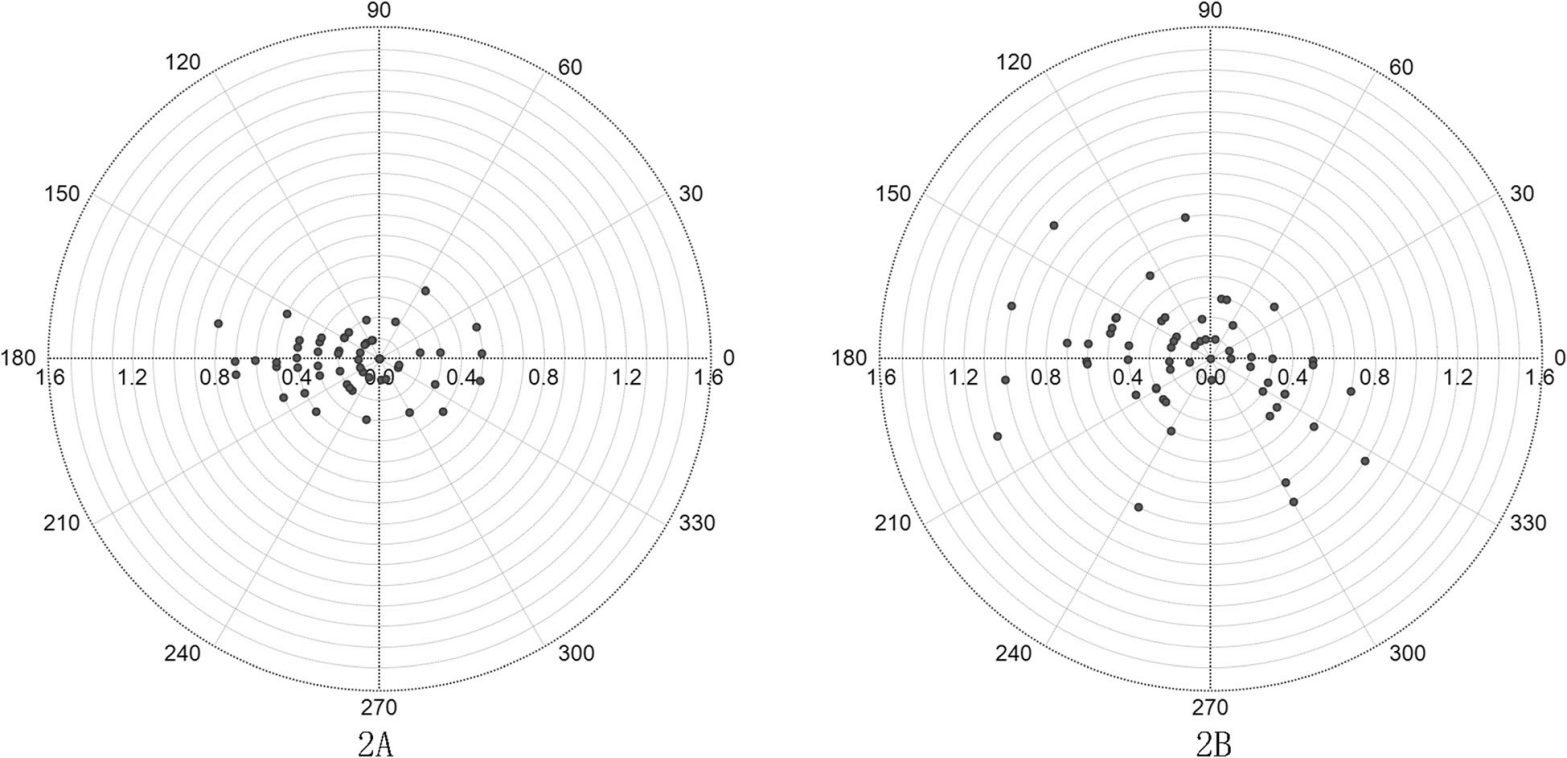
Double-angle plot of the distribution of astigmatism on the posterior corneal surface. **a** is the preoperative posterior corneal astigmatism. **b** is the postoperative posterior corneal astigmatism

The mean preoperative posterior corneal K_1_ was − 6.28 ± 0.25 D, and K_2_ was − 6.55 ± 0.27 D. The posterior corneal astigmatism ranged from 0.00 D to 0.80 D with an average of 0.28 ± 0.22 D. The postoperative posterior corneal astigmatism ranged from 0.00 D to 1.10 D with an average of 0.41 ± 0.26 D. The postoperative K_1_ was − 6.33 ± 0.28 D, and K_2_ was − 6.75 ± 0.37 D. Paired t-test showed a significant increase in postoperative posterior corneal K_1_ (*P* = 0.003), K_2_ (*P* < 0.001), and astigmatism (*P* < 0.001) compared with the preoperative one (Table 2). Figure 2 shows the preoperative and postoperative astigmatism distribution of posterior cornea. A significant decentration pattern was noted between Fig. 2a and b.

### Total corneal astigmatism

The numbers of eyes with WTR astigmatism, ATR astigmatism and oblique astigmatism of the preoperative total astigmatism corneal were 34, 23 and 3, respectively. The number of eyes with the anterior corneal astigmatism less than 1.5 D accounted for 35.0%, that of ≥1.5 D and < 2.5 D accounted for 56.7%, and that of more than 2.5 D accounted for 8.3% (Table 1).

**Fig. 3:**
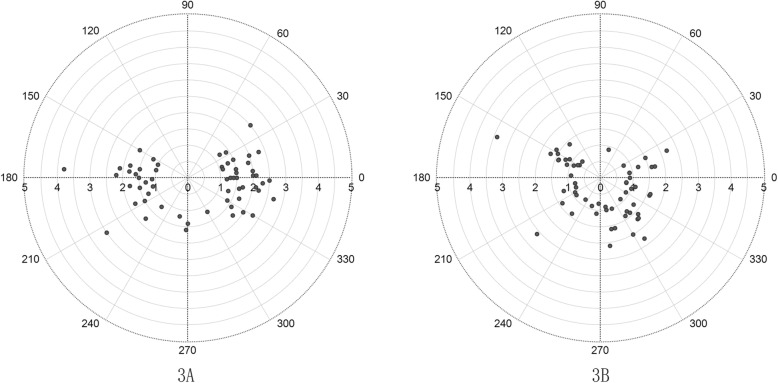
Double-angle plot of the distribution of total corneal astigmatism. **a** is the preoperative total corneal astigmatism. **b** is the postoperative total corneal astigmatism

The preoperative total corneal astigmatism ranged from 1.00 D to 3.80 D with an average of 1.70 ± 0.52 D. The postoperative total corneal astigmatism ranged from 0.75 D to 3.40 D with an average of 1.30 ± 0.51 D. Figure 3 shows the preoperative and postoperative total astigmatism distribution. A significant decentration pattern was noted between Fig. 3a and b.

### P-SIA and its correlation analysis

**Fig. 4:**
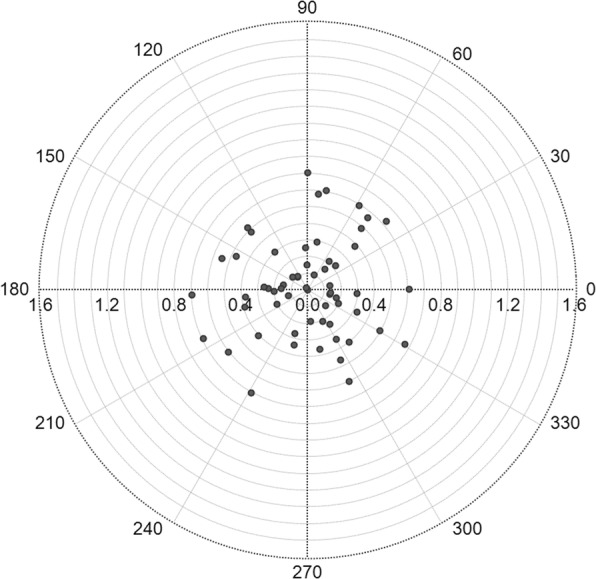
Double-angle plot of the distribution of P-SIA

P-SIA ranged from 0.00 D to 0.70 D with an average of 0.34 ± 0.20 D. There were 44 eyes with a P-SIA of less than 0.5D, accounting for 73.3%, while the P-SIA of 26.7% eyes was not less than 0.5 D (Table 3). Figure 4 reveals that P-SIA had no concentration trend in a certain direction on the polar plot.

**Table 3:** P-SIA calculated using Holladay-Cravy-Koch method

Parameters	Outcomes
Mean ± SD (D)	0.34 ± 0.20
95% CI (D)	0.29,0.39
Minimum, Maximum (D)	0.00,0.70
<0.5 D (N/%)	44 (73.3%)
≥0.5 D and<1.0 D (N/%)	16 (26.7%)
≥1.0 D (N/%)	0 (0.0%)

*CI* confidence interval, *SD* standard deviation, *P-SIA* surgically induced astigmatism on the posterior corneal, *D* diopters

According to the astigmatic axis of the posterior cornea, the corneal astigmatism was divided into three groups: WTR group (*n* = 34), ATR group (*n* = 10) and the oblique astigmatism group (*n* = 9). The P-SIA of the WTR group, ATR group and oblique astigmatism group was 0.36 ± 0.21 D, 0.38 ± 0.17 D and 0.31 ± 0.18 D, respectively. There was no significant difference among the groups by using one-way ANOVA (F = 0.335, *P* = 0.717).

The mean P-SIA of male group and female group was 0.34 ± 0.19 D and 0.34 ± 0.20 D, respectively. There was no significant difference between the groups by using Independent t-test. (*P* = 0.623).

According to the follow-up time, the corneal astigmatism was divided into three groups: 4 ~ 8w group (*n* = 15), 8 ~ 12w group (*n* = 25) and more than 12w group (*n* = 20). The P-SIA of 4 ~ 8w group, 8 ~ 12w group and more than 12w group was 0.39 ± 0.19 D, 0.35 ± 0.18 D and 0.30 ± 0.23 D respectively. There was no significant difference among the groups by using one-way ANOVA (F = 0.706, *P* = 0.498).

**Fig. 5:**
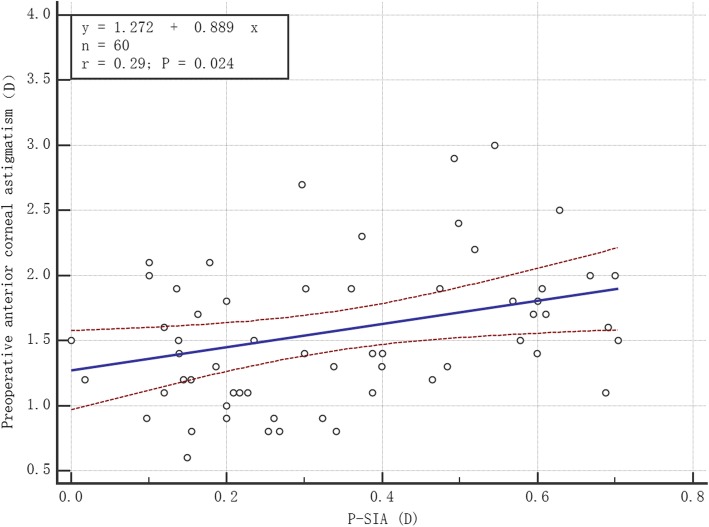
P-SIA was significantly and positively correlated with preoperative anterior corneal astigmatism (Pearson’s *r* = 0.29, *P* = 0.024). P-SIA = surgically induced astigmatism on the posterior cornea. D = diopters

**Fig. 6:**
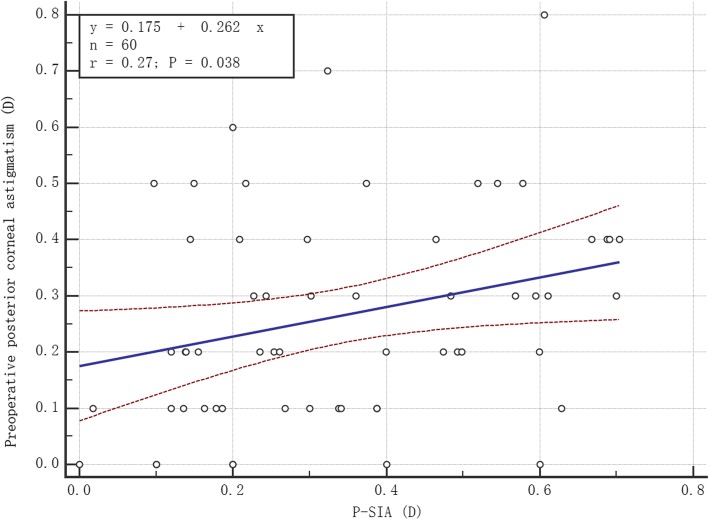
P-SIA was significantly and positively correlated with the preoperative posterior corneal astigmatism (Pearson’s *r* = 0.27, *P* = 0.038). P-SIA = surgically induced astigmatism on the posterior cornea. D = diopters

Pearson correlation analysis was performed between P-SIA and anterior/posterior corneal astigmatism, K_1_ and K_2_, and the following results were obtained. (1) P-SIA was significantly and positively correlated with anterior corneal astigmatism (*r* = 0.29, *P* = 0.024) (Fig. 5). (2) There was no significant correlation between P-SIA and anterior corneal K_1_ (*r* = 0.07, *P* = 0.576). (3) There was no significant correlation between P-SIA and anterior corneal K_2_ (*r* = 0.07, *P* = 0.608). (4) P-SIA was significantly and positively correlated with the posterior corneal astigmatism (*r* = 0.27, *P* = 0.038) (Fig. 6). (5) There was no significant correlation between P-SIA and posterior corneal K_1_ (*r* = 0.04, *P* = 0.789). (6) P-SIA was not significantly correlated with posterior corneal K_2_ (*r* = 0.18, *P* = 0.163).

In order to investigate whether the P-SIA was smaller in patients with closer axis of anterior and posterior corneal astigmatism, we performed the correlation analysis between the P-SIA and absolute axis differences of the corneal anterior and posterior astigmatism. There was no significant correlation between P-SIA and the absolute difference (*r* = 0.03, *P* = 0.808).

### Multivariable regression analysis of P-SIA

Age, sex, follow-up time, preoperative anterior corneal K_1_, K_2_ and astigmatism, preoperative posterior corneal K_1_, K_2_ and astigmatism, and the absolute axis differences of the corneal anterior and posterior astigmatism were incorporated as covariates in the multivariable regression model to adjust for their effects on P-SIA. The final multivariable model, which included preoperative anterior corneal astigmatism (B = 0.347, *P* = 0.005) and preoperative posterior corneal astigmatism (B = 0.298, *P* = 0.014), had the best-fit index with *R*^2^ = 0.205. In this multivariable model, the preoperative anterior and posterior corneal astigmatism showed a significant effect on P-SIA (F = 7.344, *P* = 0.001).

## Discussion

In recent years, the influence of posterior corneal astigmatism has caused great concern in the clinical practice with the access to the measurement of posterior corneal astigmatism. Optical coherence tomography (OCT) and Scheimpflug anterior segment analysis systems can directly measure the posterior cornea surface, which is more accurate than corneal topography systems [20, 21]. The reliability and repeatability of Pentacam have been clinically proven for corneal astigmatism measurements [22, 23].

It has been reported that the posterior cornea is a minus lens, which is WTR astigmatism [24]. With the growth of age, the meridian of posterior corneal astigmatism remains stable, while the anterior corneal astigmatism changes from WTR to ATR. If the fixed ratio is used based on the anterior corneal astigmatism, an overcorrection of 0.5 ~ 0.6 D is caused in patients with WTR astigmatism, while an undercorrection of 0.2 ~ 0.3 D is caused in patients with ATR astigmatism [10, 13].

However, it still remains controversial whether we should treat the posterior corneal astigmatism as a fixed, minus value or dynamic parameter that changes after cataract surgery. There is a debate about whether a cataract surgical incision can cause significant P-SIA. According to the results of Nemeth et al. [15], P-SIA is non-negligible with an average of 0.31 D, and about 25% of patients have a P-SIA of 0.5 D or more. In addition, it is more likely to cause lager P-SIA among patients with toric IOL implantation. Cheng et al. [14] have also found that ignoring the P-SIA may cause calculation errors of SIA. However, some studies have concluded that the effect of the incision on the posterior corneal surface is negligible. For example, Klijn et al. [16] have found that the average P-SIA is 0.1 D. Kim et al. [25] have found that P-SIA is not uniform with an average of 0.20 ± 0.17 D.

Previous studies on P-SIA [15] have predicted that P-SIA may have an important impact on the patients who are suitable for toric IOL implantation. The biomechanical effect of the incision on patients with larger corneal astigmatism may be different from those with smaller corneal astigmatism, and the accurate estimation of SIA is especially important for toric IOL implantation. Therefore, patients who are suitable for toric IOL implantation were set as the research objects in the current study. In this study, the preoperative anterior corneal astigmatism was 1.59 ± 0.69 D. Similar to previous studies [10], the posterior corneal astigmatism was 0.28 ± 0.20 D, which was dominated by WTR (62.7%).

The method described by Holladay-Cravy-Koch, which is different from simple vector calculation and has been recognized as a more accurate calculation method, was used to calculate P-SIA in this study. We found that 26.7% of patients had a P-SIA greater than 0.5 D with an average of 0.34 ± 0.20 D, suggesting that the effect of the incision on the posterior corneal astigmatism was significant and might affect the postoperative total corneal astigmatism. Besides, P-SIA has a large individual difference, possibly due to the difference in corneal rigidity, thickness, biomechanical condition and healing ability [26, 27]. The P-SIA obtained in this study was not significantly different from the P-SIA obtained by Nemeth et al. [15] (*P* = 0.64), which is larger than the P-SIA obtained by Kim et al. [25] (*P* = 0.00). This discrepancy might be attributed to the different locations of incision. This study, as well as Nemeth’s study [15], used the steepest meridian incision, while the temporal side incision is used in Kim’s study [25], suggesting that the location of the incision might have an effect on the results.

In order to explore the influencing factors of P-SIA, P-SIA was divided into different groups based on the astigmatism axis on the posterior corneal astigmatism. The results showed that there was no significant difference among the three groups, indicating that P-SIA was not associated with the astigmatism axis of the posterior cornea. The Pearson’s correlation analysis showed that P-SIA had a significant positive correlation with anterior and posterior corneal astigmatism and multivariate regression analysis also manifested that anterior and posterior corneal astigmatism had significant effect on P-SIA, which suggesting that patients with large preoperative astigmatism on the corneal anterior or posterior surface might cause larger P-SIA.

This study indicated that cataract incisions caused a significantly reduced anterior corneal astigmatism but an increase of the posterior corneal astigmatism, and the P-SIA did not show reduction trend in patients with closer axis of anterior and posterior corneal astigmatism. For anterior corneal surface, the cataract incision significantly influenced the steepest corneal meridian. For posterior corneal surface, the cataract incision significantly influenced the flattest and steepest corneal meridian. This finding could probably be attributed that the posterior corneal astigmatism was smaller than anterior corneal astigmatism, and the biomechanical characteristics of posterior corneal were different from the anterior cornea, leading to the difficulty in releasing the posterior corneal astigmatism. We speculated that the shape of the incision, corneal thickness, rigidity, especially the distance from the end of the incision to the center of the cornea, might be related to the P-SIA [28, 29]. For example, we inferred that with the shorter distance between the end of the incision and the center of the cornea, the P-SIA might be greater.

There are some advantages and significances in this study. (1) In this retrospective study, the inclusion and exclusion criteria were severely restricted to control the information bias. (2) This study considered relatively comprehensive factors that might be related to P-SIA, and we concluded that patients with larger corneal anterior or posterior astigmatism might have larger P-SIA. (3) As the accurate prediction of P-SIA of toric IOL is demanding, this study focused on patients who were suitable for toric IOL implantation and limited the corneal astigmatism of patients.

However, there are still some limitations in this study as follows. (1) The follow-up period of this study was relatively short. Although no significant difference was found among follow-up subgroups in this study and previous study has indicated that there is no significant difference of SIA between 1 month and 6 months after surgery [29], extending the follow-up time can avoid the influence of the difference in individual healing ability on the experimental results to a certain extent. (2) Although we only included the measurements with the QS reading “OK,” there might still be differences between measurement and re-measurement of the Pentacam result. (3) Some important factors, such as the distance from the end of the incision to the center of the cornea, corneal thickness and so on, were not investigated in this study due to the incomplete data. These limitations suggest that it is necessary to design a prospective, long-term study with a control group to verify the conclusion of this study.

## Conclusions

In conclusion, in candidates for toric IOL implantation with a 1.8-mm steep-axis CCI, the incision caused a significant reduction of the anterior corneal astigmatism by weakening the steep meridian, but an increase of the posterior corneal astigmatism by steepening the flat meridian and steep meridian. P-SIA could not be ignored, and it played a significant role in SIA, especially in cases with higher preoperative anterior or posterior corneal astigmatism.

## Data Availability

The datasets used and/or analyzed during the current study are available from the corresponding author on reasonable request.

## Abbreviations

CCI: Clear corneal incision
IOL: Toric intraocular lens
P-SIA: Surgically induced astigmatism on the posterior cornea
CRI: Corneal relaxing incision
LRI: Limbal relaxing incision
SIA: Surgically induced astigmatism
CCC: Continuous curvilinear capsulorhexis
WTR: With-the-rule
ATR: Against-the-rule
ANOVA: Analysis of variance
OCT: Optical coherence tomography
